# Titanium Base-Free Multi-Unit Abutment Connections: A Critical Review of Prosthetic Screw Design, Biomechanical Behavior, and Clinical Performance

**DOI:** 10.3390/ma19112212

**Published:** 2026-05-24

**Authors:** Seyed Ali Mosaddad, Iker Rodríguez-Pérez, Stefano Pieralli, Florian Beuer, Pedro Molinero-Mourelle, Gülce Çakmak

**Affiliations:** 1 Department of Conservative Dentistry and Prosthodontics, Faculty of Dentistry, Complutense University of Madrid (UCM), 28040 Madrid, Spain; ikerrodr@ucm.es (I.R.-P.); pedro.molineromourelle@unibe.ch (P.M.-M.); 2 Department of Research Analytics, Saveetha Institute of Medical and Technical Sciences, Saveetha Dental College and Hospitals, Saveetha University, Chennai 600077, India; 3 Department of Prosthodontics, Geriatric Dentistry and Craniomandibular Disorders, Campus Benjamin Franklin, Center for Dental and Craniofacial Sciences (CC3), Charité—Universitätsmedizin Berlin, Corporate Member of Freie Universität Berlin and Humboldt-Universität zu Berlin, 14197 Berlin, Germany; stefano.pieralli@charite.de (S.P.); florian.beuer@charite.de (F.B.); 4 Department of Reconstructive Dentistry and Gerodontology, School of Dental Medicine, University of Bern, 3010 Bern, Switzerland; 5 Ramon y Cajal Research Institute (IRYCIS), 28034 Madrid, Spain; 6 Department of Prosthodontics, Faculty of Dentistry, Biruni University, 34010 Istanbul, Turkey

**Keywords:** dental implants, dental prosthesis, implant-supported, dental abutments, prosthesis design, biomechanical phenomena, stress, mechanical, zirconium oxide, computer-aided design, computer-aided manufacturing, titanium base-free

## Abstract

**Highlights:**

- Titanium base-free MUA systems increase dependence on prosthetic screw biomechanics.
- Preload maintenance and screw design are key to joint stability and fatigue resistance.
- Conical and modified head screws may improve load distribution and reduce loosening.
- Rigid materials like zirconia intensify stress at the screw–abutment interface.
- Clinical success depends on passive fit, torque control, and occlusal management.

**Abstract:**

Titanium base-free multi-unit abutment (MUA) restorations have been introduced to simplify implant prosthetic workflows by eliminating intermediate titanium bases and bonding interfaces. However, this approach modifies the biomechanical behavior of the prosthesis–abutment–implant complex and increases reliance on prosthetic screw performance. Despite growing clinical and commercial interest in these systems, the available evidence remains limited and fragmented, and the biomechanical consequences of removing the titanium base have not been clearly synthesized. Therefore, this critical review evaluated the influence of prosthetic screw design on the biomechanical behavior of titanium base-free MUA restorations, focusing on preload maintenance, load transfer, and mechanical stability. The evidence indicates that preload loss, screw loosening, and fatigue behavior are primary determinants of mechanical performance. Screw material, surface characteristics, and head geometry may affect preload generation, load distribution, and resistance to micromovement, although current evidence remains limited and heterogeneous. Short-term clinical outcomes appear acceptable when appropriate biomechanical and prosthetic protocols are followed; however, long-term comparative data are lacking. Titanium base-free MUA restorations should be considered a technique-sensitive approach requiring optimized screw selection, accurate prosthetic fit, and controlled occlusal loading. Further well-designed long-term studies are needed to establish their predictability.

## 1. Introduction

Implant-supported complete-arch prostheses have become a well-established treatment option for the rehabilitation of edentulous patients, with multi-unit abutment (MUA) systems playing a central role in contemporary prosthodontics. MUAs facilitate the correction of implant angulation, improve prosthetic alignment, and support screw-retained restorations with favorable retrievability and maintenance [1]. In parallel, digital workflows have expanded the prosthetic possibilities for complete-arch implant rehabilitation, including the fabrication of monolithic zirconia fixed dental prostheses through computer-aided design and computer-aided manufacturing (CAD/CAM)-based protocols [2,3,4].

In conventional CAD/CAM workflows, these restorations frequently incorporate titanium bases as intermediary components between the prosthetic structure and the abutment. Titanium-base abutments have shown favorable short-term clinical performance, particularly in single-unit restorations, and were introduced to combine the mechanical advantages of titanium with the restorative flexibility of ceramic suprastructures [5,6]. However, the use of an intermediate titanium base also adds an additional restorative interface, commonly involving adhesive cementation, which may become a site of debonding or other technical complications. In addition, cemented implant restorations have long been considered susceptible to complications related to residual cement, although recent meta-analytic evidence suggests that cement- and screw-retained prostheses may show comparable peri-implant disease risk under controlled clinical conditions [7].

**Figure 1:**
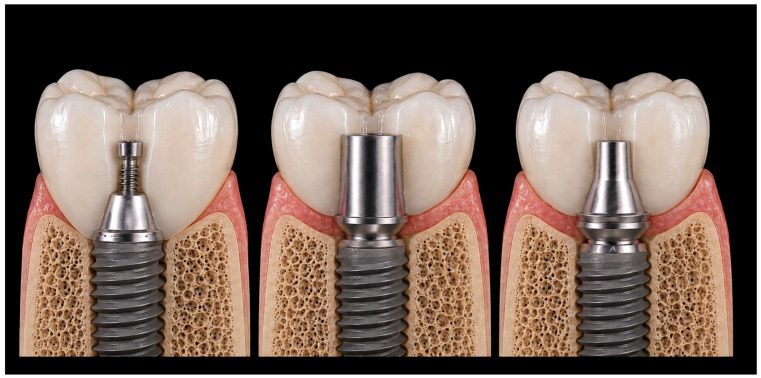
Schematic comparison of restorative connection concepts in implant-supported prostheses. (**Left**) Titanium base-free design with direct prosthesis-to-abutment connection. (**Middle**) Bar-supported design incorporating an intermediate framework. (**Right**) Conventional titanium base-supported restoration connected to a multi-unit abutment.

To reduce component interfaces and simplify restorative workflows, titanium base-free prosthetic concepts have emerged as an alternative approach in which the definitive restoration is directly connected to the MUA or implant platform, without an intermediary base. The structural differences among titanium base-free, bar-supported, and titanium base-supported configurations, particularly in terms of component interfaces and load-transfer pathways, are illustrated in Figure 1. This concept has been enabled by advances in digital workflows, manufacturing precision, and the availability of high-strength restorative materials, particularly zirconia [2,3,8]. Zirconia has become increasingly relevant in implant prosthodontics because of its favorable flexural strength, fracture resistance, and suitability for CAD/CAM fabrication, including complete-arch frameworks and monolithic reconstructions [8,9]. More recently, abutment-free or direct screw-retained prosthetic concepts have been investigated as a means of avoiding cementation-related complications and enabling a more individualized emergence profile, although they may also increase sensitivity to prosthetic fit and torque-related mechanical behavior [10].

In this context, prosthetic screws are critical not only for retention but also for maintaining joint stability through preload generation and preservation. Screw material, surface characteristics, geometry, and head design can directly influence preload, frictional behavior, load transfer, and resistance to loosening or fracture [11,12,13,14]. Experimental and numerical studies have shown that screw-head geometry affects loosening torque, preload maintenance, and stress distribution, with conical or tapered-head designs generally demonstrating improved resistance to preload loss compared with conventional flat-head screws [11,12,13,14]. Such designs are of particular interest in titanium-base-free restorations, where greater mechanical demand is transferred directly to the prosthetic screw and the prosthesis–abutment interface.

Despite the growing use of titanium base-free MUA restorations and the increasing availability of dedicated screw systems, the evidence remains limited and fragmented. Much of the current literature consists of laboratory investigations, technical descriptions, and clinical reports, whereas the biomechanical consequences of eliminating the titanium base, particularly with respect to preload maintenance, load distribution, restorative design, and long-term mechanical behavior, have not been comprehensively synthesized [2,10,14]. In addition, the specific role of prosthetic screw design in compensating for the absence of a titanium base remains insufficiently clarified.

Therefore, the purpose of this critical review is to evaluate titanium base-free multi-unit abutment connections, with particular emphasis on the influence of prosthetic screw design on biomechanical behavior, preload maintenance, restorative design considerations, and clinical performance. This review was conducted because titanium base-free MUA restorations represent an emerging prosthetic concept for which the available evidence remains limited, heterogeneous, and fragmented, despite increasing clinical and commercial interest. The specific contribution of this review is to synthesize the current biomechanical rationale for eliminating titanium bases, clarify how this design change may affect prosthetic screw behavior and load transfer, and provide a structured framework for understanding the indications, limitations, and mechanical risks of these systems. By integrating current evidence on connection mechanics, material behavior, and emerging screw concepts, this review aims to guide clinicians in the cautious application of titanium base-free MUA restorations and to encourage future standardized research on their mechanical and clinical performance.

## 2. Materials and Methods

A structured literature search was conducted to identify publications relevant to titanium base-free prosthetic concepts and prosthetic screw design in MUA connections. Electronic databases including PubMed/MEDLINE, Scopus, Web of Science, and Google Scholar were searched up to 22 March 2026.

The search strategy combined keywords and Boolean operators related to the topic, including “multi-unit abutment”, “titanium base”, “Ti-base”, “titanium base-free”, “prosthetic screw”, “abutment screw”, “preload”, “screw loosening”, “implant-supported prosthesis”, and “complete-arch restoration”. Reference lists of selected articles were also screened manually to identify additional relevant publications.

Because of the emerging nature of this topic, all study types were considered, including in vitro studies, clinical studies, case reports, technical reports, and review articles, provided they contributed relevant information on prosthetic screw behavior, connection mechanics, restorative design, or titanium base-free configurations involving MUAs. Publications focused exclusively on implant-level connections without MUA involvement or without relevance to screw mechanics were excluded.

Given the limited number of directly relevant comparative studies and the predominance of technical descriptions and early clinical reports, a narrative critical review approach was adopted rather than a formal systematic review methodology. Accordingly, studies were selected on the basis of conceptual relevance to biomechanical principles, preload behavior, restorative design considerations, and clinical performance. The objective of this review was therefore not to quantitatively synthesize outcomes, but to critically appraise the available evidence, identify recurring biomechanical relationships, and provide an integrative overview of prosthetic screw behavior in titanium base-free MUA restorations.

## 3. Concept and Classification of Titanium Base-Free MUA Connections

Titanium base-free prosthetic configurations are restorative approaches in which the definitive prosthesis is directly connected to the MUA without an intermediate titanium base. In conventional digital workflows, titanium bases are commonly used to provide a prefabricated connection geometry and to facilitate extraoral bonding between the restorative material and the abutment interface. This hybrid concept has been widely adopted because it combines the precision of a manufacturer-machined connection with the flexibility of CAD/CAM restorative design. However, in titanium base-free systems, this intermediary component is eliminated, resulting in a simplified restorative assembly in which connection stability depends primarily on prosthesis fit and the mechanical integrity of the prosthetic screw joint [15,16].

The absence of a titanium base alters the biomechanical behavior of the implant-supported reconstruction. In titanium-base-supported designs, functional loads are transferred through multiple interfaces, including the restorative material, the cement layer, the titanium base, the prosthetic screw, and the abutment [17]. By contrast, titanium base-free configurations reduce the number of interfaces and direct occlusal forces more immediately toward the MUA–screw complex. Although this simplification may reduce bonding-related complications and eliminate cementation procedures, it also increases the dependence of the system on fit accuracy, restorative material behavior, and screw design [10,15]. Abutment-free or direct screw-retained concepts have therefore been proposed to simplify the restorative complex while avoiding complications associated with cemented interfaces, although their mechanical behavior remains sensitive to torque maintenance and load transfer [10]. Titanium base-free MUA restorations can therefore be classified according to prosthetic design, prosthetic material, and fabrication technique (Table 1).

From a prosthetic design standpoint, titanium base-free MUA restorations may be broadly categorized as monolithic or framework-based. In monolithic designs, the prosthesis is fabricated as a single continuous structure, typically from a high-strength material, and connected directly to the MUAs without an intermediate base. This configuration minimizes internal material interfaces and may reduce the risk of chipping associated with veneered restorations. However, because the restoration behaves as a rigid unit, stress transmission to the prosthetic screw and abutment interface may be more pronounced, particularly when stiff materials such as zirconia are used [8,18]. Framework-based designs, by contrast, incorporate a supporting substructure directly connected to the MUAs and may be fully, partially, or veneered, depending on the restorative concept. Compared with monolithic restorations, these configurations may offer greater flexibility in esthetic layering and stress modulation, although they reintroduce additional internal interfaces within the prosthesis [8].

A second classification may be made according to prosthetic material. Titanium base-free MUA restorations have been described using materials with markedly different mechanical behaviors. Zirconia is the most widely discussed material because of its high flexural strength, fracture resistance, wear resistance, and suitability for monolithic CAD/CAM complete-arch prostheses [8]. However, its high elastic modulus may increase stress concentration at the screw joint and implant–abutment interface, particularly under nonaxial loading [8,19]. Metal-based frameworks, particularly cobalt–chromium or titanium, provide high rigidity and structural reliability, although they often require veneering and involve more complex laboratory procedures [19]. Polyetheretherketone (PEEK) and related high-performance polymers, as well as modern polymer-based and resin-composite materials, have mechanical properties that differ substantially from zirconia and metal-based frameworks. PEEK and related polymers have lower elastic moduli, which may reduce peak stress transfer to the screw–abutment interface and provide a more stress-dampening biomechanical response [18,20]. However, their lower stiffness and hardness may also increase susceptibility to deformation, wear, veneering instability, and long-term durability concerns [18,20]. Resin-based materials, including CAD/CAM resin composites, polymethyl methacrylate (PMMA), and composite-based structures, may offer advantages such as easier repairability, lower cost, shock absorption, and suitability for provisional or transitional restorations; in selected situations, they may also be used in definitive hybrid prosthetic concepts [20]. Nevertheless, their mechanical performance depends on filler content, polymerization quality, prosthetic thickness, aging conditions, and expected functional loading [20]. Because each material presents a different balance of rigidity, resilience, and fracture behavior, material selection has a direct influence on prosthetic biomechanics and the functional demands placed on the prosthetic screw [8,18,19,20]. Representative quantitative ranges for elastic modulus, hardness, strength, and fracture-related properties of these materials are provided in Supplementary Table S1.

Titanium base-free restorations may also be classified according to fabrication technique. In an all-digital workflow, impression making, prosthetic design, occlusal development, and manufacturing are performed digitally, typically through intraoral scanning, CAD design, and CAM milling or printing. This approach may improve efficiency, reduce analog steps, and facilitate direct fabrication of prostheses designed to engage the MUA without intermediary components. However, its success depends heavily on the trueness of complete-arch scanning, the accuracy of the digital library, and manufacturing precision [3,21]. In a hybrid workflow, digital and conventional laboratory procedures are combined. For example, digital planning and framework design may be followed by analog verification, conventional veneering, or laboratory finishing procedures. Although hybrid workflows may offer greater flexibility in complex complete-arch cases, they may also introduce additional sources of error through interactions between digital and analog steps [3,22].

This classification highlights that titanium base-free MUA connections do not constitute a single restorative concept but rather a group of prosthetic approaches that differ substantially in structural design, material behavior, and fabrication pathways. These differences are clinically relevant because they influence load transfer, fit accuracy, prosthetic complications, and the mechanical demands imposed on the prosthetic screw. A clear understanding of these categories is therefore essential before evaluating the specific role of screw design in the performance of titanium base-free MUA restorations [10,19].

## 4. Prosthetic Screw Characteristics in Titanium Base-Free MUA Connections

In titanium base-free MUA configurations, the prosthetic screw assumes a central biomechanical role because, in the absence of an intermediate titanium base, it becomes the principal element responsible for maintaining joint stability. In conventional titanium base-supported systems, part of the functional load may be dissipated through the base and the cement interface. By contrast, in titanium base-free restorations, occlusal forces are transferred more directly to the screw–abutment interface, increasing dependence on screw performance. Consequently, screw design, material properties, and tightening protocols become critical determinants of preload maintenance, resistance to loosening, and long-term prosthetic stability [11,12,23,24].

### 4.1. Preload Generation and Maintenance

The stability of the screw joint is governed by preload, defined as the tensile force generated within the screw during tightening. When torque is applied, the screw elongates elastically and creates a clamping force that compresses the prosthesis against the abutment. This clamping force counteracts functional loads and helps prevent separation or micromovement at the interface [10,23,24].

Only a limited portion of the applied torque is converted into preload, while most is dissipated as friction at the threads and beneath the screw head. Representative quantitative parameters related to torque-to-preload conversion, frictional loss, preload reduction, recommended torque values, and fatigue testing conditions are summarized in Supplementary Table S2. Experimental and mechanical studies have consistently shown that only about 10% to 15% of the tightening torque contributes to effective preload, whereas approximately 85% to 90% is lost in overcoming frictional resistance [23,24,25].

In titanium base-free systems, adequate preload is particularly important because the absence of an intermediate component reduces the capacity for stress buffering. Loss of preload may result in micromovement at the prosthesis–abutment interface, thereby increasing the risk of screw loosening, fatigue failure, or prosthetic fracture. Factors influencing preload include tightening torque, screw material, surface condition, lubrication, geometric design, and the precision of the mating components [12,14,24].

Preload loss may occur immediately after tightening due to the settling effect, also known as embedment relaxation [26]. This phenomenon results from flattening of microscopic surface irregularities under load, which reduces clamping force. For this reason, retightening after initial tightening has often been recommended to compensate for early preload loss and improve joint stability [24,25,27].

### 4.2. Screw Material and Surface Characteristics

The material composition of prosthetic screws plays a fundamental role in mechanical performance. Most prosthetic screws are fabricated from commercially pure titanium or titanium alloys, both of which provide favorable corrosion resistance and biocompatibility. Titanium alloys generally exhibit higher yield strength than commercially pure titanium, allowing greater preload generation without permanent deformation [11,24,28].

Surface characteristics also influence frictional behavior and, consequently, preload. Surface treatments such as gold coatings, diamond-like carbon coatings, anodization, and other lubricious finishes have been introduced to reduce friction at the thread and head interfaces. By lowering the coefficient of friction, these treatments may allow a greater proportion of the applied torque to be converted into preload, thereby improving joint stability [24,28,29].

In titanium-base-free configurations, where the screw is subjected to greater functional demands, optimization of surface properties becomes especially relevant. Reduced friction may enhance preload generation, decrease interface wear, and reduce the risk of galling or cold welding, both of which may complicate screw retrieval [24,28,29].

### 4.3. Screw Geometry and Thread Design

Screw geometry, including thread profile, pitch, diameter, and length, influences both preload generation and load distribution. A larger core diameter generally increases tensile strength and improves resistance to fracture. Thread pitch and profile affect the mechanical advantage during tightening and determine how axial forces are distributed along the screw [14,24,30].

Fine-threaded screws may allow more precise preload control, whereas coarser threads may facilitate insertion but may result in lower preload for a given torque. In addition, the extent of thread contact with the internal connection affects both frictional resistance and load transfer behavior [24,30].

In titanium-base-free systems, where load transfer is more direct, thread design becomes more important in resisting cyclic loading and fatigue. Repeated functional loading may induce micro-slippage at the thread interface, contributing to gradual preload loss and eventual screw loosening [12,14,30].

### 4.4. Screw Head Design and Load Transfer Mechanisms

The design of the screw head plays a major role in determining how forces are transferred from the prosthesis to the abutment. Conventional prosthetic screws typically have a flat or slightly tapered head that transmits load predominantly through the threaded portion. In this configuration, the screw acts mainly as a tensile element, with limited participation of the head–abutment interface in load sharing [11,12,23].

More recent designs have introduced modified head geometries, including conical and interface-engaging configurations, to alter load-transfer pathways. In these systems, the screw head seats within a corresponding geometry in the prosthesis or abutment, creating an additional contact interface. This allows part of the occlusal load to be transferred through the head–abutment interface rather than being concentrated solely within the threads [11,12,13,31].

The Rosen screw (Rosen Implant Solutions, Sherman Oaks, CA, USA) represents one example of this concept [22]. More broadly, conical- or tapered-head screw designs have been experimentally shown to improve loosening torque and preload maintenance compared with conventional flat-head screws. By increasing contact area and frictional resistance at the head interface, such designs may improve resistance to micromovement and reduce the likelihood of screw loosening, particularly in rigid prosthetic systems such as monolithic zirconia restorations [11,12,13,31].

### 4.5. Friction, Micromovement, and Screw Loosening

Screw loosening is a multifactorial phenomenon resulting from the interaction among preload, external loading, and interface friction. When functional forces exceed the preload clamping force, separation or micromovement may occur at the prosthesis–abutment interface. Repeated micromovement can progressively reduce preload and ultimately result in screw loosening [14,23,24,32].

In titanium base-free restorations, the absence of a titanium base may increase the stiffness of the prosthetic assembly, especially when high-modulus materials such as zirconia are used. This may intensify stress concentration at the screw joint and make the system more sensitive to preload loss [10,33].

Friction at the head and thread interfaces plays a dual role. Excessive friction reduces preload generation during tightening, whereas insufficient friction may reduce resistance to rotational forces during function. An appropriate balance is therefore required to maximize preload while maintaining joint stability under cyclic loading [24,29].

Modified screw designs that engage additional contact surfaces may improve resistance to loosening by increasing frictional resistance and more evenly distributing forces. However, their effectiveness remains dependent on precise manufacturing tolerances and proper torque application [11,12,13,14,31].

### 4.6. Torque Application and Clinical Protocols

The magnitude and method of torque application are critical for achieving optimal preload. Manufacturer-recommended torque values are based on the mechanical properties of the screw and connection system and should be followed carefully. Under-tightening may result in insufficient preload, whereas over-tightening may cause plastic deformation or screw fracture [23,24,25].

Torque delivery methods, including manual drivers and calibrated torque wrenches, influence the accuracy and consistency of torque application. Calibrated devices are generally preferred because they improve reproducibility and reduce operator-dependent variability, although the performance of torque-limiting devices should itself be periodically verified [27,34].

Clinical protocols often recommend retightening after a short interval to compensate for preload loss due to settling. In addition, maintaining clean and dry interfaces is important to ensure predictable frictional behavior and reliable torque-to-preload conversion [24,25,27].

### 4.7. Influence of Prosthetic Screw Geometry on Restorative Design and Manufacturability

Beyond their role in preload generation and load transfer, prosthetic screws in titanium base-free MUA restorations directly influence the geometric design, structural integrity, and manufacturability of the prosthesis. This is particularly relevant in complete-arch restorations fabricated from monolithic materials, in which the absence of a titanium base shifts both mechanical and design constraints toward the prosthesis–screw–abutment interface. In this setting, screw geometry is not only a mechanical variable, but also a key determinant of restorative feasibility and long-term structural performance [10,33,35].

One major consideration is the diameter and configuration of the screw access channel. The geometry of the screw head and driver interface directly dictates the dimensions of the access channel within the prosthesis. Systems with larger or more complex head designs may require wider channels, which can affect occlusal morphology, esthetics, and material thickness in critical regions. In monolithic zirconia restorations, screw-access preparation and channel diameter have been shown to influence fracture behavior, indicating that channel design is not merely a restorative detail but a biomechanically relevant variable [35,36].

A closely related factor is the preservation of zirconia bulk around the screw channel, particularly in monolithic restorations. In titanium base-free configurations, the access channel creates a structural discontinuity within a brittle material. When the surrounding zirconia thickness is insufficient, this area may become a stress concentrator and a potential site of crack initiation and propagation under cyclic loading. From a biomechanical perspective, maintaining adequate material bulk around the access channel is essential for minimizing peak tensile stresses and improving fatigue resistance [33,35,36].

Another important consideration is the ability to accommodate angulated screw channels, which has clear implications for both prosthetic design and clinical application. In complete-arch implant restorations, implant angulation is frequently dictated by anatomical constraints, and the ability to redirect the screw access channel may be essential for obtaining favorable emergence profiles and improved esthetics [37]. However, this capability also introduces design and biomechanical challenges, as angulation may alter the geometry of the head–abutment interface, alter the distribution of contact forces, and complicate torque delivery due to nonaxial driver engagement [37,38].

**Figure 2:**
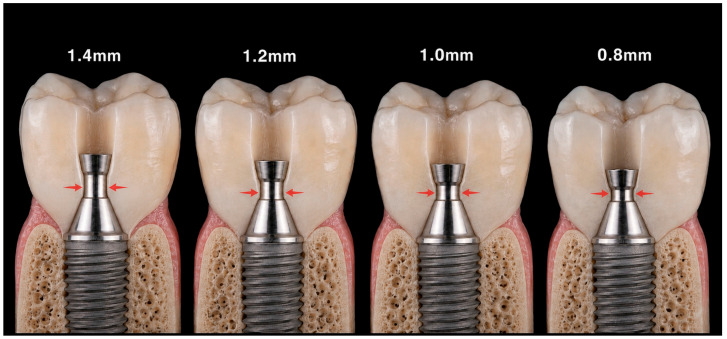
Relationship between occlusogingival restorative height and corresponding prosthetic screw length in implant-supported restorations. Representative configurations are shown with increasing restorative height and associated screw lengths (1.4 mm, 1.2 mm, 1.0 mm, and 0.8 mm), while maintaining a constant abutment interface. As restorative height increases, a longer screw segment is required, which may increase the effective lever arm during functional loading and concentrate stresses at the screw head–shank transition (arrows), a region associated with fatigue-related mechanical complications.

Finally, restorative height requirements constitute another important design constraint influenced by screw geometry. Different screw systems require different amounts of vertical space depending on head design, seating geometry, and driver engagement. When restorative space is limited, compromises may be required in either prosthetic thickness or connection design, both of which can adversely affect mechanical performance [33,35]. As illustrated in Figure 2, increased occlusogingival restorative height is associated with longer prosthetic screw configurations, which may increase the lever arm during loading and concentrate stresses at the screw head–shank junction.

Collectively, these factors demonstrate that prosthetic screw geometry plays a fundamental role in shaping both the design and the manufacturability of titanium base-free restorations. Decisions related to access-channel configuration, material thickness, angulation, and restorative space are inherently linked to screw design and must be considered during digital planning and prosthetic fabrication [10,33,35,37].

### 4.8. Implications for Titanium Base-Free Systems

In titanium-base-free MUA restorations, the prosthetic screw is not merely a retaining element but a critical component that governs the mechanical behavior of the entire system. Elimination of the titanium base shifts the biomechanical burden toward the screw–abutment interface and makes the system more sensitive to variations in screw design, preload, and material properties [10,12,33].

Accordingly, selection of an appropriate screw system, particularly one with optimized surface characteristics and head geometry, may play a decisive role in compensating for the absence of an intermediate component. In rigid prosthetic configurations, such as monolithic zirconia complete-arch restorations, these considerations become even more important because of the limited capacity of the prosthetic structure to absorb stress [10,12,33].

### 4.9. Specific Screw Systems for Titanium Base-Free MUA Restorations

**Figure 3:**
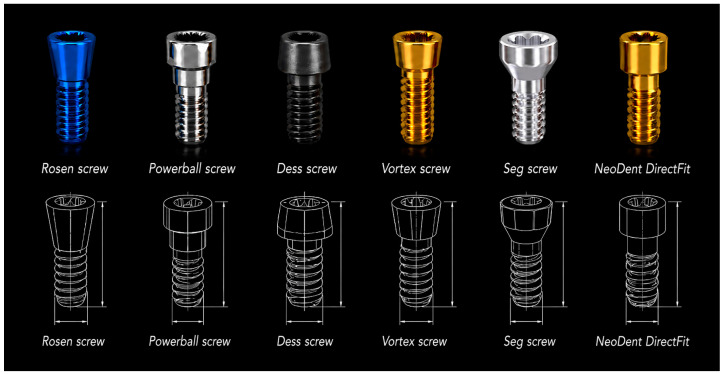
Representative prosthetic screw designs used in titanium base-free multi-unit abutment restorations. The figure illustrates variations in head geometry and overall configuration among commonly used systems. Schematic drawings (bottom row) highlight differences in head shape and dimensions, which may influence preload generation, load transfer mechanisms, and stress distribution patterns at the screw–abutment interface.

The increasing adoption of titanium-base-free MUA restorations has led to the development of specialized prosthetic screw systems that enable direct prosthesis–abutment connection without an intermediate titanium base. Unlike conventional prosthetic screws developed for earlier restorative workflows, these systems are designed to address the demands of direct load transfer, rigid restorative materials, digital complete-arch fabrication, and direct-to-MUA workflows. From a biomechanical and restorative-design perspective, currently available systems may be broadly categorized according to head geometry and load-transfer mechanism (Table 2), both of which influence preload behavior, stress distribution, seating accuracy, restorative space requirements, and manufacturability. From a conceptual perspective, these designs reflect a transition from retention-driven to biomechanics-driven screw engineering in implant prosthodontics [10,11,12,13,22,31]. Different prosthetic screw designs, characterized by variations in head geometry and seating interface, are illustrated in Figure 3, highlighting the structural differences underlying their biomechanical behavior.

#### 4.9.1. Conical-Seat Screw Systems

Conical-seat systems are among the most widely recognized categories of screws for titanium base-free restorations. These screws feature tapered or conical heads that engage a corresponding seat within the prosthesis or abutment, thereby creating an additional frictional interface that contributes to load transfer and joint stability. In general, the rationale of conical seating is to enhance frictional engagement at the head–abutment interface, improve resistance to rotational displacement, and redirect part of the functional load away from the threaded portion of the screw [11,12,13,31].

The Rosen screw (Rosen Implant Solutions LLC, Los Angeles, CA, USA) is one of the best-known systems in this category. According to the manufacturer, it features a conical head intended to engage a matching conical interface, is available in multiple diameters, including 1.4 mm, can be torqued up to 20 Ncm, and is offered in variants such as Rosen Wave and Rosen SH. Rosen also provides dedicated digital libraries for multiple CAD/CAM and photogrammetry platforms, indicating close integration with digital workflows [39,40]. From a biomechanical standpoint, the proposed rationale of this design is consistent with the broader literature on conical-head screws, which suggests that increased head–abutment contact may improve preload maintenance and reduce loosening under cyclic loading [11,12,13,31]. However, because this design relies on intimate conical seating, it may also be more sensitive to seating accuracy, insertion-path discrepancies, and framework misalignment, particularly in complete-arch prostheses where simultaneous seating across multiple units is required [11,13,31].

The Powerball screw (Xcell Dental Implant/Powerball Screw, Hawthorne, NJ, USA) represents a modified conical or radiused-seat concept. Product descriptions indicate that it features a rounded, spherical head with parallel-sided features, a head diameter of approximately 2.6 mm, and was specifically developed for direct-to-MUA complete-arch workflows [41,42]. This geometry is intended to redistribute stress away from the thread region and to facilitate CAD/CAM manufacturing by allowing top-down milling with standard burs rather than more complex undercut milling [41,42]. From a biomechanical perspective, this rationale is compatible with evidence showing that tapered or conical head designs may improve torque maintenance and reduce stress concentration compared with flat-head screws, although direct peer-reviewed comparative data for the Powerball system itself remain lacking [11,12,13,31]. The larger head geometry may also require greater restorative height and a wider access channel, which may reduce surrounding prosthetic material thickness in space-limited situations [41,42].

The Vortex screw (Louisiana Dental Implant Lab/Vortex, Lafayette, LA, USA) is another system marketed for direct-to-MUA workflows, particularly in complete-arch restorations. Commercial sources indicate that the Vortex system is available in diameters of 1.4 mm and 1.8 mm, typically has a maximum torque of 15 Ncm, and is distributed with exocad- and 3Shape-compatible digital files [43,44]. Although peer-reviewed biomechanical evidence for this system remains limited, the design appears to follow a conical or hybrid-seat concept intended to improve load distribution and facilitate restorative workflows [43,44]. Conceptually, it may be regarded as an intermediate design between strongly tapered systems and more rounded geometries, aiming to balance retention, stress modulation, and restorative bulk. Nonetheless, in cases with steep implant angulation or limited tool access, milling complexity may remain a relevant limitation [43,44].

Collectively, conical-seat systems are intended to enhance frictional engagement at the head–abutment interface, improve preload stability, and redistribute functional forces. Their performance, however, remains highly dependent on manufacturing accuracy, because minor discrepancies in seating geometry may lead to incomplete seating or uneven load distribution, particularly in complete-arch prostheses. This issue may be especially relevant in multi-implant frameworks, in which conical designs inherently rely on simultaneous centering across multiple abutments, and therefore may be less tolerant of minor divergence in implant angulation, screw-channel preparation, or framework fit [11,13,31].

#### 4.9.2. Flat-Seat Screw Systems

Flat-seat systems have emerged as an alternative design philosophy to address some of the limitations of conical seating in titanium base-free restorations.

The most representative example is the DESS Flat Seat Screw (DESS Dental Smart Solutions/Terrats Medical S.L., Barberà del Vallès, Barcelona, Spain), which was developed specifically for direct-on-MUA prosthetic applications. Unlike conical designs, this system uses a flat, planar head–abutment interface that allows more uniform contact and reduces reliance on precise angular seating. According to DESS, the system is associated with a torque value of approximately 15 Ncm, may be provided in DLC-coated versions, and supports angulated screw channels up to 25 degrees when used with the company’s digital libraries [45]. The manufacturer also emphasizes a thicker seating platform and greater tolerance to direct-on-MUA restorative workflows [45].

From a biomechanical standpoint, the flat-seat design may allow a greater proportion of the applied torque to be converted into preload by reducing frictional variability associated with tapered interfaces. In addition, the broader contact surface may promote more uniform load distribution. This rationale is mechanically plausible, especially in the context of multi-unit frameworks where simultaneous seating accuracy is critical, but independent comparative evidence specifically validating the superiority of flat-seat systems over conical alternatives in long-term clinical conditions is still limited [11,14,45].

DESS has also highlighted potential limitations of conical-seat designs, including sensitivity to milling accuracy, variability in final seating height, and possible axis misalignment [45]. The flat-seat concept may therefore be interpreted as an attempt to improve seating predictability and tolerance to minor misfit rather than relying primarily on mechanical interlocking. In multi-unit frameworks, this design philosophy is particularly relevant because it may reduce the risk that minor deviations in implant trajectory or screw-channel preparation generate internal tension during prosthesis delivery. Nevertheless, the supporting evidence remains largely manufacturer-driven, and independent comparative studies are still needed to determine whether flat-seat designs offer consistent biomechanical advantages over conical alternatives under long-term clinical conditions [14,45].

The Neodent DirectFit Screw (Neodent/Straumann Group, Basel, Switzerland) represents another contemporary flat-seat direct-to-MUA concept. According to manufacturer information [46], this system uses a flat seating surface with a beveled transition intended to create a controlled contact interface and reduce sensitivity to manufacturing tolerances across different milling systems. The design also incorporates a self-centering effect, an extended occlusal shaft intended to facilitate control and seating through long or angulated channels, and a Ti6Al4V-ELI alloy body with a recommended installation torque of 20 Ncm. The system is promoted for direct screw-retained bridges and complete-arch restorations on Neodent mini-conical and micro-conical abutments and is integrated with open digital libraries compatible with exocad DentalCAD software/digital libraries (exocad GmbH, Darmstadt, Germany; https://exocad.com), 3Shape Dental System software/digital libraries (3Shape A/S, Copenhagen, Denmark; https://www.3shape.com), and CARES Visual software (Institut Straumann AG, Basel, Switzerland; https://www.straumann.com). Conceptually, this design aligns with the broader flat-seat philosophy by prioritizing seating tolerance, even force distribution, and digital workflow integration. However, as with other recently introduced proprietary systems, the available support is currently derived primarily from manufacturer documentation, and independent comparative biomechanical and clinical evidence remains limited.

#### 4.9.3. Rounded or Radiused Head Designs

Rounded or radiused head designs represent a variation in conical systems with greater emphasis on stress redistribution than on geometric locking.

The Powerball screw (Xcell Dental Implant/Powerball Screw, Hawthorne, NJ, USA) may also be considered within this category because of its spherical head geometry, which promotes more even force distribution within the prosthesis [41,42]. This characteristic is particularly relevant in monolithic zirconia restorations, where sharp stress concentrations around the screw channel may predispose to crack initiation or catastrophic fracture. The rationale for this design is consistent with the broader transition from conventional straight-walled screw seats toward smoother, stress-modulating geometries better suited to brittle monolithic materials [12,31,35].

By increasing contact surface area and smoothing the transition between the screw head and the prosthetic material, radiused designs aim to reduce localized stress peaks and improve fatigue resistance. In addition, these geometries may facilitate simplified milling with larger burs, thereby reducing manufacturing complexity and improving laboratory efficiency in direct-to-MUA workflows [41,42]. However, the potential advantages of these systems must be weighed against their greater restorative-space requirements, because larger or more rounded seat configurations may necessitate a wider channel or greater vertical height, thereby affecting material thickness in critical prosthetic regions [35,41,42].

This category reflects a broader shift toward stress-management-driven design, particularly in monolithic zirconia complete-arch restorations, in which the screw-seat region may act as a critical site for stress concentration and crack initiation [35].

#### 4.9.4. Alternative Head Geometry and Retention-Enhancing Designs

More recent systems have introduced additional geometric features intended to improve retention, stability, and compatibility with digital workflows. The SegScrew (SegMark Workflow, Cypress, TX, USA) is a representative example. According to official product information, it incorporates anti-rotational grooves beneath the head, a tapered neck of approximately 30 degrees, and a head diameter of approximately 2.4 mm, while also supporting angulated screw channels up to 30 degrees, TorX-compatible drivers, and digital workflow integration [47]. These features are intended to improve rotational resistance, enhance vertical clamping, and facilitate milling of the screw channel using larger-diameter burs [47].

The Badger Screw (Smart Mouth Technologies, Bozeman, MT, USA) represents another alternative concept. Official descriptions state that it features a concave head profile intended to enhance clamping force, reduce prosthetic stress, and permit a smaller screw channel [48]. This concept differs from both conical and flat-seat approaches by optimizing clamping behavior and stress transfer through a distinct head profile rather than planar seating or taper-based engagement. Although the biomechanical rationale is plausible, current support remains largely manufacturer-driven, and independent peer-reviewed evidence remains limited [48].

Another emerging concept is the matrix SmartBolt/Tri Matrix screw (TRI Dental Implants Int. AG, Hünenberg, Switzerland). TRI Dental Implants describes its matrix^®^ connection as a digital implant interface specifically designed for modern manufacturing workflows, and company materials note that the matrix^®^ SmartBolt includes different screw-head configurations intended to support material-specific milling strategies [49]. However, independent evidence specifically validating biomechanical or clinical advantages of this concept remains very limited.

Across all currently available categories, the common objective is to compensate for the absence of a titanium base by improving preload stability, load distribution, resistance to micromovement, and compatibility with contemporary digital complete-arch workflows. At the same time, it is important to recognize that most contemporary developments in this field are primarily driven by manufacturer innovation and digital workflow requirements rather than robust comparative clinical evidence. Although the biomechanical rationale for these systems is plausible, direct comparisons among different screw designs remain scarce. No single system can therefore be considered universally superior, because clinical performance depends on multiple interacting factors, including prosthetic material, framework design, occlusal loading, implant distribution, restorative space, and manufacturing accuracy. For this reason, selection of a screw system should be based on a comprehensive biomechanical and clinical assessment rather than on design features or manufacturer claims alone [11,12,13,14,31].

#### 4.9.5. Force-Based Classification of Direct-to-MUA Prosthetic Screws

In addition to classifying prosthetic screws according to head geometry and seating configuration, direct-to-MUA screw systems may also be categorized according to the dominant force generated during tightening and functional loading. A recently proposed practical classification [4] describes four main mechanical design philosophies: flat compression, frictional wedging, cradle-based vertical capture, and locking anti-rotational mechanics (Table 3). This approach is useful because it relates screw design directly to the direction and distribution of applied forces at the prosthesis–screw–abutment interface. However, because this classification is currently derived mainly from educational and manufacturer-based sources, it should be interpreted as a conceptual framework rather than as an evidence-based hierarchy of clinical performance.

Type 1 screws, or flat compression screws, generate primarily vertical clamping forces. These screws have a flat-bottom seating surface that compresses the prosthesis against a defined seating ledge. Their biomechanical behavior is passive because they do not create lateral wedging or compensate for misalignment. Therefore, their performance depends strongly on prosthetic passivity, accurate CAD/CAM fabrication, and precise internal seating geometry. In cases of framework misfit, the flat compression interface may reveal the discrepancy rather than compensate for it, potentially increasing stress concentration in brittle restorative materials such as zirconia [4].

Type 2 screws are based on frictional wedging mechanics and generate both downward and outward forces. These screws engage the internal walls of the prosthesis through a tapered interface, creating frictional locking that may resist micromovement, rotation, and vertical displacement. Short-wedge designs, classified as Type 2a, use a shorter taper and may be easier to mill, more compatible with thinner prosthetic walls, and less aggressive in terms of lateral stress. Long-wedge designs, classified as Type 2b, use extended taper engagement to increase rigidity and mechanical locking, but they may require thicker restorative walls and more precise machining to avoid excessive internal stresses [4].

Type 3 screws, or cradle screws, rely on vertical capture rather than flat compression or wedging. In this concept, the screw engages a rounded or U-shaped seat that distributes forces over a broader contact surface. This configuration is intended to reduce localized stress concentration and provide controlled seating, particularly in provisional materials, printed resins, PMMA restorations, and selected definitive applications. The broad contact area may be advantageous when controlled stabilization is preferred over aggressive frictional locking [4].

Type 4 screws incorporate locking anti-rotational mechanics. These systems are designed not only to generate axial clamping force but also to resist reverse rotation and screw back-out. This concept is particularly relevant in low-torque or non-elongation screw mechanics, where resistance to rotational loosening may become a key determinant of long-term stability. Some designs use additional thread geometry or mechanical anti-rotational features within the prosthetic channel to reduce the tendency for reverse rotation during function [4].

**Figure 4:**
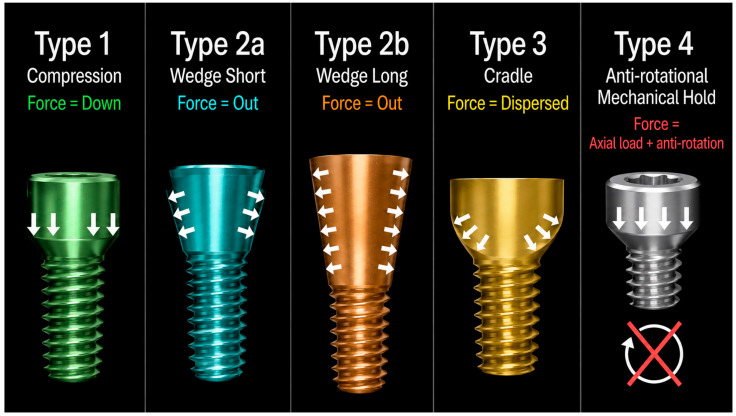
Force-based classification of direct-to-MUA prosthetic screw designs. Type 1 flat-compression screws generate primarily vertical clamping forces directed apically against a flat seating surface. Type 2a short-wedge and Type 2b long-wedge screws generate outward force through frictional engagement with the prosthetic walls, with the long-wedge design providing a greater contact area and more extensive lateral engagement. Type 3 cradle screws distribute forces over a broader rounded seating surface, allowing dispersed load transfer and vertical capture. Type 4 anti-rotational screws combine axial clamping with a mechanical locking effect intended to resist reverse rotation and screw back-out. Arrows indicate the direction of dominant force application, and × symbols indicate areas where rotational locking or resistance to screw back-out is generated.

Overall, this force-based classification [4] complements geometry-based classifications by emphasizing how each screw design applies load to the prosthetic assembly. Flat compression screws primarily generate vertical clamping; wedge screws add lateral frictional engagement; cradle screws distribute forces through broad vertical capture; and locking screws combine axial loading with anti-rotational stability (Figure 4). Nevertheless, direct comparative studies are still needed to determine whether these theoretical force-application differences translate into measurable improvements in preload maintenance, fatigue resistance, screw loosening, or long-term clinical outcomes.

To further relate the force-based classification to currently available commercial systems, a product-level comparison can be summarized according to seat type, compatible restorative materials, vertical adjustability, angulated screw-channel compatibility, driver system, manufacturer, FP1 applicability, and recommended torque (Table 4) [4]. These data provide a practical overview for clinicians and technicians; however, because they are derived from educational/manufacturer-based information, they should be interpreted cautiously and should not be considered evidence of comparative clinical superiority. Independent biomechanical and long-term clinical studies remain necessary to validate the performance of these systems under standardized conditions.

## 5. Biomechanical Considerations in Titanium Base-Free MUA Connections

The biomechanical behavior of titanium base-free MUA restorations is determined by the interaction among prosthetic design, restorative material, abutment geometry, and prosthetic screw characteristics. Elimination of the titanium base reduces the number of restorative interfaces but simultaneously alters the pathway through which functional forces are transferred across the prosthesis–abutment–implant complex. As a result, the system becomes more directly dependent on the integrity of the screw joint and the precision of the prosthesis–MUA interface. This shift is biomechanically relevant because even minor discrepancies in fit, preload loss, or stress concentration may have amplified consequences in rigid, directly connected restorations [10,14,27,31].

In conventional titanium base-supported restorations, the intermediate titanium base may contribute to stress modulation by acting as a metallic transition zone between the restorative material and the abutment. Although this additional interface may introduce technical complications, such as debonding or cement-related problems, it may also partially modify force transmission by interposing a ductile component between the prosthesis and the supporting connection. In titanium base-free systems, this transition zone is absent. Occlusal loads are therefore transferred more directly from the restorative material to the MUA and prosthetic screw, which may increase tensile, compressive, and shear stresses at the screw–abutment interface. Consequently, biomechanical tolerance depends more heavily on prosthetic passivity, material stiffness, screw preload, and connection geometry [14,27,50].

A central concept in these restorations is the relationship between joint stability and external loading. The prosthetic screw generates a clamping force through preload, and as long as functional loads remain below this clamping force, the joint behaves as a stable unified structure. However, when cyclic occlusal loads approach or exceed this threshold, microseparation may occur at the interface, initiating micromovement and progressive preload loss. In titanium base-free restorations, this process may be more critical because the direct connection provides less opportunity for interface accommodation. Once clamping integrity is compromised, the screw is exposed to greater bending and shear stresses, which may accelerate loosening or fatigue failure [11,12,13,23].

Prosthetic fit is another decisive biomechanical variable. Passive fit has long been regarded as essential in implant prosthodontics because implants lack the periodontal ligament and therefore exhibit minimal physiologic mobility. Any framework misfit may introduce static strain even before functional loading begins. In titanium base-free restorations, where the prosthesis engages the MUA directly, a lack of passivity may generate internal stresses that are transferred immediately to the screw joint and abutment interface. This is especially relevant in complete-arch restorations, where small inaccuracies may accumulate across multiple implants and create distortive forces during screw tightening. Under these conditions, the screw may function not only as a retaining element but also as a compensatory mechanism attempting to draw a misfitting prosthesis into place, thereby reducing effective preload and increasing the risk of mechanical complications [50,51].

The elastic modulus of the restorative material also exerts a major influence on stress distribution. Materials with a high modulus of elasticity, such as zirconia and cobalt–chromium, resist deformation under load and therefore transmit a larger proportion of occlusal forces directly to the supporting interfaces. Although this may be advantageous in terms of structural rigidity and resistance to bulk deformation, it also increases stress concentration at the screw joint and implant–abutment connection. By contrast, materials with a lower elastic modulus, such as high-performance polymers or resin-based structures, may absorb part of the functional load through elastic deformation, thereby reducing peak stress transmission to supporting components. This potential biomechanical advantage, however, may be offset by reduced long-term dimensional stability, greater wear, or increased susceptibility to veneering failure. Thus, material selection is not merely a matter of strength; it determines whether the prosthesis behaves predominantly as a rigid stress-transmitting unit or as a more resilient stress-dampening assembly [14,52,53,54].

Among the available materials, monolithic zirconia deserves particular attention because it is frequently proposed for direct-to-MUA complete-arch prostheses. Its favorable flexural strength, fracture toughness, wear resistance, and suitability for digital manufacturing make it attractive for titanium base-free applications. Nevertheless, its high stiffness limits its ability to dissipate functional loads, especially under off-axis or cantilevered loading. In a titanium base-free configuration, this may intensify stress at the screw head, threads, and abutment interface, particularly if the prosthesis is not fully passive or if occlusal contacts are not carefully controlled. For this reason, the biomechanical performance of zirconia-based titanium base-free restorations is closely linked to accurate digital workflows, careful occlusal design, and optimized screw geometry [8,14,52,55].

The prosthetic design itself also influences biomechanical behavior. Monolithic restorations behave as continuous structures and eliminate internal prosthetic interfaces, thereby reducing the risk of chipping or delamination. However, their rigidity may cause the prosthesis to distribute forces broadly across the arch while simultaneously concentrating stress at the most constrained interfaces, particularly the screw–abutment junction. Framework-based designs may behave differently depending on whether they are fully anatomical, cutback, or blended configurations. A rigid metallic framework may distribute forces efficiently across multiple implants, whereas frameworks combined with veneering composites or lower-modulus overlay materials may display a more complex combination of rigidity and resilience. In blended or partially anatomical designs, different regions of the prosthesis may respond differently to functional loading, thereby altering both bulk stress distribution and the type of force transmitted to the screw joint [52,53,54].

A related consideration is the presence or absence of cantilevers. Cantilever extensions increase bending moments and amplify nonaxial loading, thereby increasing tensile and shear stresses on distal implants, abutments, and screws. In titanium base-free systems, this effect may be particularly unfavorable because the direct restorative connection reduces the number of stress-interrupting interfaces. As a result, cantilevers may magnify the biomechanical disadvantages of high-stiffness materials and increase the likelihood of screw loosening, fracture, or ceramic complications. Conversely, minimizing or eliminating cantilevers generally improves the mechanical prognosis of the prosthetic complex [1,53,54,56,57,58].

The direction and magnitude of occlusal forces are equally important. Implant-supported prostheses are biomechanically most favorable when forces are directed axially, because axial loading tends to be distributed more uniformly along the implant body and supporting bone. Lateral and oblique forces generate bending moments and asymmetric stress distribution, particularly at the crestal bone and connection level. In titanium base-free restorations, where direct load transfer predominates, nonaxial forces may more readily destabilize the screw joint and increase micromovement at the interface. This is particularly relevant in complete-arch prostheses, where parafunctional activity, uneven occlusal contacts, or poorly controlled excursions may generate repeated eccentric loading. Accordingly, occlusal design is not merely a finishing step but a major determinant of connection stability [50,52,53,54,58].

Patient-related clinical conditions may further modify the biomechanical risk profile of titanium base-free MUA restorations. Bruxism and other parafunctional habits can generate repeated high-magnitude and nonaxial forces, increasing the risk of preload loss, micromovement, screw loosening, prosthetic fracture, and fatigue-related complications [50,52,53,54,58]. These effects may be particularly relevant in titanium base-free configurations because occlusal forces are transferred more directly to the prosthetic screw–abutment interface, especially when high-stiffness materials such as zirconia are used [10,14,52,55]. Bone quality may also influence mechanical stability, as compromised implant support can increase micromovement and alter load distribution, thereby increasing stress at the prosthetic connection and surrounding peri-implant bone [50,51]. Therefore, patients with bruxism, compromised bone quality, unfavorable implant distribution, or high occlusal loading should be considered higher-risk candidates for titanium base-free MUA restorations.

The role of angulated MUAs must also be considered. Multi-unit abutments are frequently used to compensate for implant angulation and facilitate screw-retained prosthetic access, particularly in complete-arch rehabilitations [59]. Although MUAs improve restorative alignment, the use of angulated abutments may alter force direction and increase bending moments under function, depending on the degree of angulation and prosthetic design. In titanium base-free systems, these effects may be more pronounced because the prosthesis is connected directly to the abutment without an intermediary titanium base that might otherwise influence contact geometry and force transmission [59]. When angulated MUAs are combined with rigid restorative materials and long-span prostheses, the importance of precise fit and stable screw preload becomes even greater. At the same time, angulated abutments may also enable more favorable implant distribution and wider support zones, which can reduce cantilever effects in complete-arch designs [1,37,54].

Another crucial issue is fatigue behavior under cyclic loading. Implant-supported restorations are subjected to repeated functional loads over prolonged periods, and most mechanical complications arise not from single overload events but from the cumulative effect of cyclic stress. Even when the applied load is below the ultimate strength of the screw or restorative material, repeated cycles may cause progressive damage through fatigue mechanisms. In titanium base-free restorations, fatigue risk may increase if preload decreases over time, because reduced clamping force permits micromovement and increases local stress concentration within the screw. Likewise, rigid materials that transmit load more directly may subject the screw to repeated high-magnitude stress cycles, particularly during eccentric function. Therefore, long-term biomechanical success depends not only on initial fit and torque, but also on the ability of the screw–abutment interface to maintain stability under repeated loading [11,12,13,23].

The screw head–abutment interface has emerged as an especially important biomechanical zone in this context. Conventional screw designs rely mainly on the threads to maintain clamping and resist functional loads. By contrast, modified screw geometries with conical or interface-engaging heads may introduce additional frictional contact and a broader load-bearing area at the head–abutment interface. This concept is particularly relevant in titanium base-free restorations because it may partially compensate for the absence of an intermediate metallic base by improving force distribution across the connection. Although direct comparative evidence remains limited, the biomechanical rationale for such designs is strong, particularly in rigid zirconia restorations where thread overloading and micromovement are major concerns [11,12,13,35].

From a broader perspective, the biomechanical performance of titanium base-free MUA restorations should be understood as the result of component interaction rather than isolated behavior. A favorable screw design cannot fully compensate for severe framework misfit, excessive cantilever length, inappropriate occlusal scheme, or unsuitable restorative material. Similarly, an accurately fitting prosthesis may still be vulnerable if preload is inadequate or if the screw geometry is poorly suited to the loading conditions. This interdependence is particularly important when interpreting the literature, because studies often vary simultaneously in prosthetic material, design, tightening protocol, implant distribution, abutment angulation, and method of fit verification. For this reason, biomechanical analysis of titanium base-free systems must remain integrative rather than reductionist [14,23,52,54].

Overall, titanium base-free MUA restorations offer theoretical advantages in terms of fewer interfaces, simplified workflows, and elimination of titanium base–related complications. However, these benefits are achieved at the cost of greater mechanical dependence on the direct prosthesis–abutment connection. As a result, the system becomes more sensitive to preload loss, fit inaccuracies, material stiffness, and unfavorable occlusal loading. Successful application of titanium base-free concepts therefore requires meticulous control of prosthetic passivity, restorative design, material selection, occlusal scheme, and screw characteristics. These biomechanical principles provide the basis for understanding both the potential benefits and the limitations of this restorative approach and are essential when interpreting its mechanical and clinical performance (Table 5) [14,23,31,52,58].

## 6. Mechanical and Clinical Outcomes in Titanium Base-Free MUA Connections

The mechanical and clinical performance of titanium base-free MUA restorations is determined by the combined influence of prosthetic design, material properties, fit accuracy, and, critically, prosthetic screw behavior. Because titanium base-free concepts have only recently gained broader clinical attention, the available evidence remains heterogeneous and is derived predominantly from laboratory investigations, technical reports, and a limited number of clinical observations. Accordingly, interpretation of the literature requires caution, because many reported advantages remain supported more strongly by biomechanical rationale than by long-term comparative clinical evidence. Nevertheless, several recurring trends can be identified with respect to preload maintenance, screw stability, fatigue behavior, restorative complications, and short-term clinical performance [2,60,61,62,63,64].

### 6.1. Preload Loss and Reverse Torque Values

Preload maintenance is a key determinant of screw-joint stability and is commonly assessed through reverse torque value measurements after tightening and cyclic loading (Table 6). In general, preload loss occurs immediately after tightening as a result of embedment relaxation and may continue progressively during functional simulation. In titanium base-free configurations, this phenomenon may be particularly consequential because elimination of the titanium base reduces the number of interfaces capable of modifying stress transfer and places greater mechanical dependence on the screw–abutment complex. Under these conditions, even modest loss of preload may permit micromovement at the prosthesis–abutment interface and thereby initiate a cascade leading to rotational instability, screw loosening, or fatigue damage [64,65].

The available evidence suggests that reverse torque behavior is influenced not only by the magnitude of the applied torque, but also by screw surface condition, lubrication, head geometry, and framework fit. Screws with reduced friction coefficients, such as coated or lubricated designs, may achieve higher initial preload for a given torque and may therefore show improved reverse torque values after loading. Likewise, head geometries that increase contact area at the screw–abutment interface may reduce micro-slippage and improve preload retention [6,64,65]. However, these findings should be interpreted carefully, because reverse torque values obtained under laboratory conditions do not necessarily translate directly into clinical superiority. The mechanical relevance of improved preload retention depends on whether it is sufficient to preserve joint integrity under the more complex and variable loading conditions encountered intraorally [64,65].

### 6.2. Screw Loosening and Joint Stability

Screw loosening remains one of the most frequent technical complications in implant-supported prosthodontics and is generally regarded as a consequence of insufficient preload, adverse loading conditions, or instability at the connection interface [60,64,66]. In titanium base-free restorations, the risk of loosening may be amplified by the rigidity of the restorative assembly, particularly when high-modulus materials such as monolithic zirconia are used. Because these materials undergo limited elastic deformation under load, a greater proportion of functional stress may be transferred directly to the screw joint. This may increase the likelihood that cyclic loads approach or exceed the effective clamping force, thereby promoting microseparation and progressive preload loss [60,61,64].

Laboratory studies consistently support the concept that cyclic loading, particularly under nonaxial conditions, increases susceptibility to screw loosening [64,65,67]. This effect is especially relevant in long-span or complete-arch restorations, where cantilevers, implant divergence, and uneven occlusal contacts may generate complex bending moments [2,60,62,63]. In titanium base-free systems, such forces may be transmitted more directly to the screw–abutment interface because no intermediary titanium base is present to alter contact mechanics. Modified screw designs, especially those incorporating conical or interface-engaging heads, have therefore been proposed as a means of improving joint stability by redistributing part of the functional load away from the threads and increasing frictional resistance at the head interface [6,64,65]. Although the biomechanical rationale is persuasive, direct clinical evidence demonstrating lower loosening rates for one specific design over another remains limited. At present, screw loosening should be understood primarily as a multifactorial complication reflecting the interaction among preload, fit, material stiffness, and occlusal loading rather than as a simple consequence of screw geometry alone [60,64,65].

### 6.3. Fatigue Resistance and Fracture Behavior

Fatigue resistance is a major determinant of long-term mechanical performance because most technical failures in implant-supported prostheses arise under cyclic rather than static loading. In titanium base-free restorations, fatigue behavior reflects the cumulative interaction among screw preload, prosthetic passivity, restorative material stiffness, and loading direction. High-strength materials such as zirconia may resist bulk fracture effectively, but their rigidity may also result in repeated transfer of relatively high stresses to the screw and abutment interface [61,64,67]. If preload is reduced over time, the screw may become increasingly exposed to bending and shear stresses, thereby accelerating fatigue crack initiation and propagation [64,65,67].

From a mechanical perspective, screw fracture is seldom an isolated event; rather, it is often the end-stage consequence of prior instability within the joint. Progressive loosening increases micromovement, alters stress concentration within the screw, and may shift loading from predominantly tensile to more damaging bending-dominated patterns [64,65]. For this reason, fatigue failure in titanium base-free systems should not be interpreted solely in terms of ultimate screw strength, but also in terms of the system’s ability to preserve clamping integrity over time. Framework-based or lower-modulus restorative designs may reduce peak stress transmission to the screw complex, potentially improving fatigue behavior at the connection level. However, this apparent advantage may be offset by greater susceptibility to other complications, such as veneering fracture, delamination, or wear. Thus, fatigue performance depends less on any single component than on the mechanical balance of the restoration as a whole [61,67,68].

### 6.4. Passivity, Fit Accuracy, and Their Mechanical Implications

Passive fit remains essential for the long-term success of implant-supported restorations, but its significance may be even greater in titanium base-free systems because the prosthesis is connected directly to the MUA without an intermediate compensatory component [65,69]. Any misfit may introduce static stress during screw tightening, effectively consuming part of the available preload before functional loading even begins [70]. Under these conditions, the screw is forced to act not only as a retaining element but also as a corrective mechanism attempting to seat a nonpassive prosthesis, thereby reducing joint stability and increasing susceptibility to loosening or fracture [65,69].

Although digital workflows have improved manufacturing precision, complete-arch accuracy remains challenging, particularly when intraoral scanning is used. Errors related to scan-body capture, stitching, digital library alignment, manufacturing tolerance, or post-processing may accumulate across multiple implants and become clinically relevant in complete-arch restorations [65,69,70,71,72,73]. For this reason, claims regarding the predictability of titanium base-free workflows must be interpreted in light of the persistent gap between digital precision and true mechanical passivity. Clinical reports suggest that passive titanium base-free restorations can be achieved when digital workflows are combined with careful verification methods such as the one-screw test, visual inspection for absence of rocking, and radiographic confirmation. However, these observations should not be interpreted as evidence that passive fit is inherently easier to achieve in direct-to-MUA restorations; rather, they emphasize that favorable outcomes depend on strict validation of fit at delivery [2,63,65,73].

### 6.5. Material-Related Mechanical Complications

The prosthetic material influences not only the likelihood of restoration-related complications, but also the nature of the loads transmitted to the screw–abutment interface. Monolithic zirconia restorations are attractive in titanium base-free applications because of their high flexural strength, wear resistance, and compatibility with digital complete-arch workflows [2,60,61,63,64]. At the same time, their high elastic modulus may intensify stress concentration at the screw joint, particularly around the screw channel and head interface. As a result, the mechanical success of zirconia-based titanium base-free restorations depends heavily on adequate prosthetic thickness, careful channel design, passive fit, and controlled occlusal loading [60,61,63,74].

Framework-based restorations incorporating veneering or lower-modulus materials may provide a different biomechanical profile. By allowing some degree of elastic deformation or stress redistribution, they may reduce peak force transmission to the supporting interfaces. However, this potential benefit must be interpreted against their increased vulnerability to chipping, delamination, wear, or long-term surface degradation [61,68,74]. Similarly, polymer-based and hybrid restorative materials may offer greater shock absorption, but their lower stiffness does not automatically confer superior clinical performance, because excessive deformation or material fatigue may itself become a source of complication. Accordingly, material-related outcomes in titanium base-free restorations should not be framed simply as a comparison between “rigid” and “resilient” materials, but rather as a trade-off between connection stress, prosthetic durability, and maintenance burden [61,68,74].

### 6.6. Clinical Outcomes and Reported Complications

Clinical evidence on titanium base-free MUA restorations remains limited, but the reports currently available suggest that favorable short- to medium-term outcomes are achievable when these systems are used under controlled conditions [2,22,60,62,63]. Reported complications are broadly similar to those observed in implant-supported complete-arch prostheses more generally and include screw loosening, prosthetic fracture, occlusal wear, and the need for maintenance adjustments [60,61,62,63,64]. Importantly, the absence of frequent catastrophic complications in early clinical reports should not be overinterpreted as proof of long-term predictability, because most published reports involve limited follow-up and highly selected workflows [2,60,62,63].

Published clinical observations describing monolithic zirconia prostheses directly connected to MUAs with dedicated screw systems generally report stable implants, acceptable prosthetic performance, and high patient satisfaction over short observation periods [2,62,63]. These favorable outcomes are usually associated with careful case selection, minimization of cantilever extension, verification of passive fit, and controlled occlusal design [2,60,62,63]. This pattern is clinically meaningful because it suggests that success is less likely to depend on the mere absence of a titanium base than on the extent to which biomechanical risk factors are proactively controlled. In other words, current clinical evidence supports cautious feasibility rather than definitive superiority [2,60,62].

### 6.7. Comparison with Titanium Base-Supported Systems

Compared with titanium base-supported restorations, titanium base-free systems offer several theoretical and practical advantages, including reduced component complexity, elimination of adhesive cementation procedures, and more streamlined digital workflows [2,6]. These features may reduce technique sensitivity and remove complications associated with bonding failure or cement remnants. However, the apparent simplification of the restorative assembly should not be confused with reduced biomechanical complexity. On the contrary, removal of the titanium base shifts greater mechanical responsibility to the direct prosthesis–abutment connection and to the prosthetic screw itself [6,64,65].

Titanium base-supported systems may benefit from the presence of an additional interface that can act as a metallic transition zone and, in some restorative configurations, provide a degree of stress modulation or restorative flexibility. Titanium base-free systems, by contrast, rely more heavily on precise fit, stable preload, accurate machining, and appropriate material behavior to maintain joint integrity during function (Table 7) [6,64,65,74]. At present, the available evidence does not conclusively establish the superiority of either approach. Instead, it indicates that titanium base-free restorations may perform favorably when biomechanical principles are rigorously respected, whereas titanium base-supported restorations may remain preferable in situations where restorative geometry, bonding reliability, or mechanical tolerance favors the presence of an intermediate component [2,6,64].

## 7. Advantages and Limitations of Titanium Base-Free MUA Connections

Titanium base-free MUA restorations have emerged as an alternative to conventional prosthetic workflows, with potential advantages related to simplification of the restorative assembly and elimination of bonding interfaces. However, these potential benefits are accompanied by biomechanical and technical challenges that are closely linked to the behavior of the prosthetic screw and the direct nature of the prosthesis–abutment connection. A balanced appraisal of these advantages and limitations is essential for appropriate clinical application [3,10,15].

### 7.1. Advantages

One of the principal advantages of titanium base-free systems is the reduction in prosthetic interfaces. By eliminating the titanium base and its associated cement layer, the number of junctions within the restorative complex is reduced. This may decrease the risk of complications related to adhesive failure, including debonding, cement degradation, and contamination associated with residual cement. In addition, removal of the bonding interface eliminates variability related to surface treatment protocols, adhesive selection, and cementation technique, thereby reducing technique sensitivity [3,10,15].

A second potential advantage is the simplification of the digital workflow. Titanium base-free restorations can be designed and fabricated within a digital environment, allowing direct engagement of the prosthesis with the MUA connection geometry [3,21]. By eliminating titanium-base selection, surface treatment, extraoral bonding, cementation, and bonding-interface verification, this approach may reduce certain laboratory and clinical steps, shorten production time, and facilitate integration with technologies such as virtual articulation and digitally guided occlusal design [3,21]. Such streamlining may be particularly advantageous in complete-arch rehabilitations, where workflow efficiency and reproducibility are clinically relevant [3,21].

From a biomechanical standpoint, elimination of an intermediate component creates a more direct load transfer pathway. When prosthetic fit and occlusion are properly controlled, this may improve the predictability of force transmission. In this context, optimized screw designs, particularly those that enhance head–abutment engagement, may further improve load distribution and joint stability by partially compensating for the absence of a titanium base [6,10,50].

Titanium base-free systems may also offer potential advantages in terms of component reduction and cost efficiency, as they eliminate the need for prefabricated titanium bases and certain associated laboratory procedures. This may be especially relevant in extensive rehabilitations or in workflows designed to maximize digital efficiency [16,21].

### 7.2. Limitations

Despite these advantages, titanium base-free MUA restorations present several important limitations, most of which arise from their increased biomechanical sensitivity [10,50,64].

A major limitation is the greater dependence on prosthetic screw performance. In the absence of a titanium base, the screw–abutment interface becomes the principal site for load transfer and joint stability. Consequently, any deficiency in preload, screw geometry, surface behavior, or tightening protocol may directly compromise the system’s integrity. Although modified screws with conical or interface-engaging geometries may improve load distribution and resistance to loosening, robust clinical evidence supporting their superiority remains limited [10,50].

Another important limitation is the increased sensitivity to prosthetic misfit. Because the prosthesis is directly connected to the MUA without an intermediary component, inaccuracies in fit may cause internal stresses during screw tightening. These stresses may reduce effective preload, increase the risk of loosening, and contribute to mechanical complications over time. Achieving passive fit remains particularly challenging in complete-arch restorations, where cumulative errors may occur during complete-arch data acquisition and prosthetic manufacturing [3,65].

The mechanical behavior of restorative materials also represents a limitation. High-modulus materials such as zirconia, which are commonly used in titanium base-free restorations, transmit occlusal forces more directly to the supporting interfaces. Although these materials provide excellent strength and wear resistance, their rigidity reduces the system’s capacity to absorb functional loads and may increase stress concentration at the screw joint and implant–abutment interface, particularly under nonaxial loading [8,10,50,64].

A further limitation is the scarcity of long-term clinical evidence. Most currently available data are derived from laboratory studies, technical reports, or short-term clinical observations. Although these reports suggest that favorable outcomes are possible when the systems are properly executed, the absence of long-term controlled studies limits definitive conclusions regarding reliability, complication rates, and comparative performance relative to titanium base-supported restorations [5,10,15,64].

Certain titanium base-free designs may also show reduced reparability. This is particularly relevant for monolithic complete-arch restorations, in which localized repair of fracture, wear, or esthetic defects may be more limited than in veneered or modular prosthetic designs. In rigid monolithic zirconia prostheses, corrective intervention may require more extensive adjustment or remake [8,64].

Finally, titanium base-free systems may be more sensitive to occlusal design and functional loading conditions. Because forces are transmitted more directly to the screw–abutment interface, unfavorable occlusal schemes, cantilever extensions, or uncontrolled lateral forces may increase the risk of mechanical complications. This underscores the importance of careful occlusal planning, particularly in complete-arch restorations fabricated from rigid materials [50,64].

### 7.3. Overall Considerations

The potential advantages of titanium base-free MUA restorations are primarily related to simplification, reduction in interfaces, and improved integration with digital workflows. These benefits, however, are counterbalanced by increased biomechanical sensitivity to fit, material behavior, occlusal loading, and screw performance. Accordingly, titanium base-free restorations should not be regarded as a universal replacement for titanium base-supported systems, but rather as a technique-sensitive alternative that may offer advantages when applied under appropriate clinical conditions and with careful attention to biomechanical principles [3,10,15,50,64].

## 8. Clinical Recommendations for Titanium Base-Free MUA Restorations

Successful implementation of titanium base-free MUA restorations requires strict adherence to biomechanical principles because these systems are highly dependent on prosthetic screw performance and prosthesis–abutment interface accuracy. The following recommendations are based on the currently available evidence and biomechanical rationale and are intended to optimize clinical outcomes while minimizing mechanical complications [1,11,12,13].

### 8.1. Case Selection and Indications

Titanium base-free restorations are most appropriate in clinical situations in which accurate digital workflows and controlled biomechanical conditions can be achieved. Complete-arch rehabilitations with favorable implant distribution, adequate anteroposterior spread, and minimal cantilever extension represent the most suitable indications. Under these conditions, load distribution across multiple implants may reduce the mechanical burden on individual screw joints [1,52,53].

By contrast, cases involving excessive cantilever length, unfavorable implant angulation, or high parafunctional activity should be approached with caution. In such scenarios, increased bending moments and nonaxial loading may amplify stress at the screw–abutment interface and exceed the biomechanical tolerance of titanium base-free systems [1,53,54].

### 8.2. Selection of Prosthetic Screw Systems

Given the central role of the prosthetic screw in titanium base-free restorations, screw selection should be regarded as a primary determinant of treatment success rather than a secondary component choice.

Screws with optimized material properties and surface characteristics, such as titanium alloys with reduced-friction coatings, may facilitate higher preload generation and improved preload retention. In addition, designs incorporating conical or interface-engaging head geometries may offer biomechanical advantages by redistributing functional loads and increasing resistance to micromovement [10,11,12,13]. In rigid prosthetic configurations, particularly monolithic zirconia complete-arch restorations, the use of such dedicated screw designs may be especially beneficial for compensating for the absence of a titanium base and reducing stress concentration at the screw joint [10,11,12,13].

### 8.3. Torque Protocols and Preload Optimization

Strict adherence to manufacturer-recommended torque values is essential for achieving optimal preload without inducing plastic deformation or fracture of the screw. The use of calibrated torque devices is recommended to improve reproducibility and reduce operator-dependent variability [12,27,34].

A re-tightening protocol should also be considered to compensate for embedment relaxation. Re-torquing after an initial settling period may help restore preload and improve joint stability. In addition, maintaining clean and uncontaminated interfaces during screw insertion is critical for predictable frictional behavior and reliable torque-to-preload conversion [12,34,75].

### 8.4. Prosthetic Fit and Verification

Achieving passive fit is a fundamental requirement in titanium base-free restorations. Because the prosthesis directly engages the MUA, any misfit may generate internal stresses that compromise preload and increase the risk of mechanical complications [13,65,73]. Verification protocols such as the one-screw test, clinical assessment for absence of rocking, and radiographic evaluation should be routinely used before definitive prosthesis delivery. In full-arch restorations, where cumulative inaccuracies may occur, additional verification steps, including try-in prostheses, may further improve accuracy.

Although digital workflows offer high precision, clinicians should remain aware of their limitations, particularly in complete-arch scanning, and incorporate appropriate validation strategies [65,73].

### 8.5. Material Selection and Prosthetic Design

Material selection should be guided by a balance between mechanical strength and stress distribution characteristics. High-strength materials such as zirconia provide excellent durability, but their high stiffness may increase stress concentration at the screw–abutment interface. In these situations, careful occlusal control and appropriate screw selection become particularly important [1,11,64].

Framework-based or hybrid prosthetic designs incorporating materials with lower elastic modulus may provide improved stress modulation, although they also introduce additional interfaces and their own potential complications. The choice between monolithic and framework-based designs should therefore be individualized according to biomechanical demands, esthetic requirements, and maintenance considerations [1,52,64].

### 8.6. Occlusal Design and Load Control

Occlusal scheme is a major determinant of the biomechanical performance of titanium base-free restorations. Axial loading should be prioritized because it promotes more uniform stress distribution and reduces bending moments at the implant–abutment interface [1,50,54,62].

Nonaxial forces, including lateral and eccentric contacts, should be minimized through careful occlusal adjustment. In complete-arch restorations, mutually protected occlusion or controlled canine guidance may help reduce the magnitude and frequency of nonaxial loading [62,76].

The incorporation of digital occlusal analysis or jaw-motion tracking systems may further improve occlusal accuracy and reduce the need for extensive chairside adjustments, particularly in complex rehabilitations [22,76].

### 8.7. Management of Cantilevers and Implant Distribution

Minimizing or eliminating cantilever extensions is strongly recommended in titanium base-free restorations. Cantilevers increase bending moments and amplify stress at distal implants and screw joints, particularly in rigid prosthetic systems [53,54,62].

Optimizing implant distribution, including maximizing anteroposterior spread and ensuring adequate support in load-bearing regions, may reduce the biomechanical burden on individual components and improve overall system stability [53,54].

### 8.8. Maintenance and Follow-Up

Regular follow-up is essential for monitoring the mechanical performance of titanium base-free restorations. Clinical evaluation should include assessment of screw stability, occlusal contacts, prosthesis integrity, and radiographic monitoring of peri-implant bone levels [63,66].

Early detection of complications, such as preload loss or screw loosening, allows timely intervention and may prevent progression to more severe mechanical failures, including screw fracture or prosthetic damage [63,66].

### 8.9. Overall Clinical Perspective

Titanium base-free MUA restorations represent a promising approach that may simplify workflows and reduce component-related complications. However, their successful use requires a high level of technical precision and biomechanical control, with particular emphasis on screw selection, preload optimization, prosthetic passivity, and occlusal management [1,11,12,13,62,65].

When these factors are carefully controlled, titanium base-free systems may achieve predictable outcomes. Conversely, neglect of these principles may increase the risk of mechanical complications (Table 8), underscoring the importance of a mechanically informed and protocol-driven clinical approach [1,63,64,66].

## 9. Future Perspectives

Titanium base-free MUA restorations represent an evolving concept in implant prosthodontics, and several areas require further investigation to clarify their long-term mechanical and clinical predictability [10,14].

One of the most important priorities is the optimization of prosthetic screw design specifically for titanium base-free applications. Many currently available screw systems have been adapted from conventional implant or titanium base-supported workflows, and their performance in direct-to-MUA prosthetic assemblies remains insufficiently characterized. Future developments may focus on screw geometries that improve load distribution, such as refined conical interfaces, multi-contact head designs, and hybrid frictional-mechanical locking concepts. Advances in surface engineering, including low-friction coatings and wear-resistant treatments, may also enhance preload generation and long-term stability under cyclic loading [10,14].

Another key area is the interaction between restorative material behavior and connection mechanics. The relationship between high-stiffness materials, such as zirconia, and the screw–abutment interface warrants further investigation, particularly under nonaxial and fatigue loading conditions. Development of functionally graded materials or hybrid prosthetic designs that combine rigidity in load-bearing regions with resilience in less critical areas may help achieve a more favorable biomechanical response and reduce stress concentration at vulnerable interfaces [10,14].

Further progress is also expected from digital workflows and manufacturing accuracy. Improvements in intraoral scanning, photogrammetry, and data-processing algorithms may enhance complete-arch accuracy and facilitate more predictable passive fit in titanium base-free restorations [3,71]. In addition, artificial intelligence–assisted design and occlusal optimization may allow more individualized adjustment of prosthetic contours and contact patterns based on patient-specific functional data [77].

From a clinical research perspective, there is a clear need for well-designed comparative studies evaluating titanium base-free and titanium base-supported systems under standardized conditions. Long-term clinical trials assessing screw stability, complication rates, prosthesis survival, and maintenance requirements are particularly needed. Comparative studies of different prosthetic screw designs, including conventional and modified geometries, will also be essential for validating their proposed biomechanical advantages in titanium base-free restorations [10,14].

Finally, broader clinical adoption will depend on the development of standardized protocols for screw selection, torque application, prosthetic verification, occlusal design, and maintenance. As material science, digital dentistry, and connection design continue to evolve, titanium base-free systems may become a more predictable treatment option, provided that their biomechanical limitations are more clearly understood and appropriately managed.

## 10. Conclusions

Titanium base-free MUA restorations represent an emerging prosthetic concept that simplifies implant-supported restorative workflows by eliminating intermediate titanium bases and bonding interfaces. However, this simplification increases the biomechanical dependence of the restoration on the direct prosthesis–abutment interface and prosthetic screw joint. The available evidence indicates that preload maintenance, screw loosening, micromovement, and fatigue behavior are the main mechanical factors influencing the performance of these systems. Screw design, surface characteristics, prosthetic fit, restorative material stiffness, and occlusal loading all contribute to load transfer and joint stability.

Modified screw designs may improve force distribution and resistance to loosening, but current evidence is insufficient to identify one design as clinically superior. Therefore, titanium base-free MUA restorations should be considered technique-sensitive and should be used with careful control of screw selection, torque application, passive fit, restorative thickness, cantilever extension, and occlusion. Although short-term clinical feasibility has been reported, long-term comparative data remain limited. Future standardized laboratory, finite element, fatigue, and clinical studies are needed to quantify mechanical performance and establish predictable indications for these restorations.

## Figures and Tables

**Table 1:** Classification of titanium base-free multi-unit abutment prosthetic configurations.

Classification Level	Category	Description	Examples	Biomechanical Implication
Prosthetic Design	Monolithic	Single-piece prosthesis directly connected to MUA	Monolithic zirconia full-arch	High rigidity → increased stress on screw
	Framework-based	Substructure with or without veneering	Co-Cr + composite, PEEK frameworks	Improved stress distribution but more interfaces
Material Type	Zirconia	High-strength ceramic	Monolithic complete-arch zirconia	High modulus → stress concentration
	Metal-based	Co-Cr frameworks	Hybrid prostheses	High rigidity, predictable strength
	Polymer-based	PEEK, PMMA, composites	Interim or hybrid prostheses	Stress-damping but lower durability
Fabrication Technique	Fully digital	CAD/CAM workflow	Milled zirconia, printed resin	High precision, workflow efficiency
	Hybrid workflow	Digital + analog steps	Veneered frameworks	More flexible but more variables

**Table 2:** Comparison of prosthetic screw systems for titanium base-free multi-unit abutment restorations.

Screw System	Manufacturer	Design Category	Head/Seat Geometry	Key Mechanical Features	Angulation Capability	Typical Torque Range	Main Advantages	Potential Limitations
Rosen Screw	Rosen Implant Solutions LLC, Los Angeles, CA, USA	Conical-seat	Conical/tapered head engaging matching seat	Load transfer via head–abutment interface; increased frictional engagement; reduced thread stress	Up to ~30° (Wave/SH variants)	~20 Ncm	Improved preload stability; reduced micromovement; small screw head; strong digital integration	High sensitivity to seating accuracy; potential misfit-related internal stress and retrieval difficulty in complete-arch cases
Powerball Screw	Xcell Dental Implant/Powerball Screw, Hawthorne, NJ, USA	Radiused/conical hybrid	Spherical/rounded head (~2.6 mm)	Stress redistribution away from threads; smoother load transfer; milling-friendly geometry; top-down milling with standard burs	Up to ~20°	~15–20 Ncm	Reduced stress concentration in zirconia; simplified CAD/CAM manufacturing; direct-to-MUA workflow facilitation	Limited independent clinical data; greater restorative height and wider access channel may compromise prosthetic bulk in space-limited cases
Vortex Screw	Louisiana Dental Implant Lab/Vortex, Lafayette, LA, USA	Conical/hybrid	Tapered or hybrid seating design	Designed for direct-to-MUA workflows; digital library compatibility; simplified prosthetic connection	Limited (varies by system)	~15 Ncm	Workflow efficiency; compatibility with complete-arch restorations; balance between retention and restorative bulk	Limited published biomechanical evidence; hybrid seating may still require precise alignment and complex milling in angulated cases
DESS Flat Seat Screw	DESS Dental Smart Solutions/Terrats Medical S.L., Barberà del Vallès, Barcelona, Spain	Flat-seat	Planar/flat head interface	Uniform seating; reduced dependence on milling precision; improved preload consistency; DLC coating options	Limited (depending on system; angulated channels supported in some configurations)	~15 Ncm	Improved seating predictability; tolerance to minor misfit; consistent preload; favorable for complete-arch zirconia workflows	Less reliant on geometric interlocking; long-term comparative evidence versus conical systems remains limited
Neodent DirectFit Screw	Neodent/Straumann Group, Basel, Switzerland	Flat-seat	Flat seating surface with beveled transition	Controlled contact interface; self-centering effect; extended 0.8 mm shaft; even force distribution; Ti6Al4V-ELI alloy; digital-library integration	Compatible with straight and angulated mini-conical abutments; brochure shows use up to 60° in some indications	20 Ncm	Improved seating tolerance; support for long/angled channels; strong digital workflow integration; direct-to-MUA full-arch applicability; reduced coping-related steps	Evidence mainly manufacturer-derived; system-specific compatibility; possible restorative-space/material-thickness constraints; independent long-term comparative data lacking
SegScrew	SegMark Workflow, Cypress, TX, USA	Retention-enhancing/hybrid	Enlarged head (≈2.4–2.6 mm) with grooves; tapered interface (~30°)	Anti-rotational grooves; enhanced vertical clamping; large head for stress distribution and milling ease	Yes (angulated channels supported)	~15–20 Ncm	Improved rotational stability; workflow optimization; compatible with larger burs	Limited peer-reviewed validation; design complexity
Badger Screw	Smart Mouth Technologies, Bozeman, MT, USA	Alternative head geometry	Concave head profile	Concave head geometry intended to enhance vertical clamping, reduce stress concentration, and preserve prosthetic bulk through a smaller screw channel	Not clearly defined	~15–20 Ncm (reported)	Stress reduction in zirconia; smaller channel may improve esthetics and preserve material bulk	Limited published data; unclear long-term performance
Tri Matrix Screw	TRI Dental Implants Int. AG, Hünenberg, Switzerland	Mixed flat-rounded concept	Flat-bottom and rounded-top geometry	Intended combination of seating tolerance and stress redistribution	Not clearly defined	Not clearly defined	Alternative design concept aiming to balance load distribution and seating behavior	Very limited independent evidence; uncertain long-term clinical relevance

**Table 3:** Force-based classification of direct-to-MUA prosthetic screws and corresponding systems.

Type	Design Philosophy	Dominant Force Application	Mechanical Behavior	Corresponding Screw Systems/Brands	Typical Restorative Implications
Type 1—Flat compression	Flat-bottom compression	Purely vertical clamping force	Passive compression against a flat seating ledge; no lateral wedging or pull	DESS Flat Seat Screw 19.018/19.069; DESS 19.098; SIN PRH 30/40 screw	Requires precise prosthetic passivity and accurate internal geometry; may increase stress in brittle materials when fit is imperfect
Type 2a—Short wedge	Frictional wedging	Downward force with moderate outward engagement	Short taper creates frictional locking with reduced lateral stress compared with long-wedge designs	Rosen SH short screw; SegScrew	More forgiving, easier to mill, suitable for thinner prosthetic walls and angulated access channels
Type 2b—Long wedge	Frictional wedging	Downward force with strong outward engagement	Extended taper maximizes mechanical locking, rigidity, and wall engagement	Rosen screw; Rosen Wave screw	Provides high stability but requires thicker restorative walls and highly precise machining
Type 3—Cradle	Cradle-based vertical capture	Broadly dispersed force over a rounded or U-shaped seat	Vertical capture with broad contact area and controlled force distribution	Vortex screw; PowerBall screw; Zirkonite/PowerBall variant	Favorable for PMMA, printed provisionals, hybrid temporaries, and selected definitive restorations where reduced localized stress is desirable
Type 4—Locking anti-rotational	Wedge-based seat with anti-rotational locking mechanics	Axial load with resistance to reverse rotation	Mechanical anti-rotational features reduce the tendency for screw back-out	Badger Screw	Intended to improve rotational stability, particularly in low-torque or mechanically demanding direct-to-MUA situations

**Table 4:** Commercial direct-to-MUA screw systems according to force-based classification and technical characteristics.

Direct-to-MUA Screw System	Force-Based Class	Seat Type	Material Compatibility	Adjustable Vertical Position	ASC Compatibility	Driver Type	Manufacturer	FP1 Applicability	Recommended Torque
DESS 19.018/19.069 Flat Seat Screw	Type 1	Flat	C, T, Z	1–2 mm adjustment	No/25°	0.050/1.27 mm hex	DESS, Dental Smart Solutions/Terrats Medical S.L., Barberà del Vallès, Barcelona, Spain	Yes	15 Ncm
DESS 19.098 Flat Seat Screw	Type 1	Flat	C, T, Z	1–2 mm adjustment	No	Straumann Torx driver	DESS Dental Smart Solutions/Terrats Medical S.L., Barberà del Vallès, Barcelona, Spain	No	15 Ncm
PRH 30/40 Screw	Type 1	Flat	C, T, Z	Yes	No	0.050/1.27 mm hex	SIN Implant System, São Paulo, SP, Brazil	Yes	10–20 Ncm
Rosen SH Short Screw, 1.4, 1.6, and 1.72 mm	Type 2a	Wedge	R, P, T	1–2 mm adjustment	20°	T6	Rosen Implant Solutions LLC, Los Angeles, CA, USA	No	20 Ncm
SegScrew	Type 2a	Wedge	Z, R, P, T	Yes	30°	TorX6	SegMark Workflow, Cypress, TX, USA	Yes	15 Ncm
Rosen Screw/Rosen Wave Screw	Type 2b	Wedge	R, P, T	1–2 mm adjustment	20°	T6/1.25 mm hex driver	Rosen Implant Solutions LLC, Los Angeles, CA, USA	No	20 Ncm
Vortex Screw, 1.4, 1.6, and 1.72 mm	Type 3	Cradle	Z, R, C, P, T	0.8–1.4 mm adjustment	25°	T5 driver	LA Dental Implant Lab/Vortex, Lafayette, LA, USA	Yes	15 Ncm
PowerBall Screw	Type 3	Cradle	Z, R, C, P, T	1–3 mm adjustment	30°	Not available	Xcell Dental Implant/PowerBall Screw, Hawthorne, NJ, USA	No	25 Ncm
Badger Screw	Type 4	Wedge/locking anti-rotational	Z, R, P	No	20°	1.2 mm/0.048-inch hex	Smart Mouth Technologies, Bozeman, MT, USA	Yes	15 Ncm for resin-based restorations; 20 Ncm for zirconia

C—composite; T—titanium; Z—zirconia; R—resin; P—PMMA; ASC—angulated screw channel; FP1—fixed prosthesis type 1.

**Table 5:** Biomechanical implications of prosthetic screw design in titanium base-free multi-unit abutment restorations.

Design Category	Representative Systems	Preload Behavior	Load Transfer Mechanism	Stress Distribution Pattern	Sensitivity to Misfit	Performance in Rigid Materials (e.g., Zirconia)	Risk of Screw Loosening	Clinical Implication
Conical-seat	Rosen, Vortex	Moderate to high (dependent on seating accuracy)	Dual mechanism: thread + head–abutment conical interface	More evenly distributed between threads and head interface	High (requires precise simultaneous seating in multi-unit frameworks)	Favorable if seating is accurate; may concentrate stress if misfit exists	Moderate (reduced if properly seated)	Suitable for high-load cases with precise digital workflows and controlled fit
Radiused/spherical head	Powerball	Moderate to high	Load redistribution through rounded head geometry; reduced thread dependence	Reduced stress concentration at screw channel; smoother stress gradients	Moderate	Highly favorable; may reduce crack initiation risk in zirconia	Moderate to low	Preferred in monolithic zirconia restorations where stress concentration around the screw channel is a concern
Flat-seat	DESS Flat Seat Screw; Neodent DirectFit Screw	More consistent preload/seating behavior, less dependent on conical wedging	Primarily thread-based with broad, uniform head contact	More uniform vertical load distribution; reduced sensitivity to localized seating discrepancies	Lower than conical-seat concepts, though still dependent on framework fit	Favorable when adequate prosthetic thickness is maintained; potentially advantageous in direct-milled monolithic full-arch restorations	Moderate	Advantageous in complete-arch digital workflows where seating predictability, tolerance to minor discrepancies, and access through long or angulated channels are important, although independent evidence remains limited.
Retention-enhancing/hybrid	SegScrew	Moderate to high	Combined mechanisms: thread + head + anti-rotational features	Distributed load with improved rotational resistance	Moderate	Favorable; enhanced stability under cyclic loading	Low to moderate	Suitable for complex complete-arch restorations requiring rotational stability and digital workflow adaptability
Concave/alternative head geometry	Badger Screw	Potentially favorable (limited evidence)	Enhanced vertical clamping through concave interface	Reduced localized stress; potential stress redirection away from prosthesis	Moderate (data limited)	Potentially favorable; designed to reduce zirconia stress	Unknown (limited data)	Promising for esthetic zones and stress-sensitive restorations, but requires further validation
Mixed flat-rounded concept	Tri Matrix Screw	Uncertain	Intended combination of planar seating and rounded stress modulation	Theoretically reduced localized stress with improved seating tolerance	Uncertain	Potentially favorable, but insufficient evidence	Unknown	Emerging concept with theoretical advantages, but currently lacking validation

**Table 6:** Factors influencing preload and stability of prosthetic screw joints.

Factor	Effect on Preload	Mechanism	Clinical Consequence
Screw material	↑ preload with higher-strength alloys	Higher yield strength allows greater elastic elongation	Improved joint stability
Surface treatment (e.g., DLC, coating)	↑ preload	Reduced friction at threads/head	Reduced loosening risk
Head geometry	Variable	Load transfer through head vs threads	Affects stress distribution
Torque application	Directly proportional	Higher torque → higher preload (within limits)	Under/over-tightening risks
Settling effect	↓ preload	Surface micro-flattening	Requires re-torque
Prosthetic misfit	↓ effective preload	Screw compensates misfit	Higher loosening/fracture risk
Occlusal loading	↓ preload over time	Cyclic micro-movement	Fatigue and loosening

↑ indicates an increase or favorable effect; ↓ indicates a decrease or unfavorable effect; → indicates causing, leading to, or resulting in the indicated outcome.

**Table 7:** Comparison of titanium base-free and titanium base-supported prosthetic systems.

Parameter	Titanium Base-Supported	Titanium Base-Free
Interfaces	Multiple (prosthesis–cement–Ti-base–abutment)	Reduced (direct prosthesis–abutment)
Load transfer	More distributed	More direct
Screw dependence	Moderate	High
Risk of debonding	Present	Eliminated
Sensitivity to misfit	Moderate	High
Workflow	More steps	Simplified digital workflow
Repairability	Easier (modular)	More limited (monolithic)
Biomechanical risk	Moderate	Higher (if poorly controlled)

**Table 8:** Mechanical complications in titanium base-free restorations and their underlying mechanisms.

Complication	Primary Cause	Role of Screw Design	Prevention Strategy
Screw loosening	Preload loss, cyclic loading	Head geometry affects stability	Proper torque, screw selection
Screw fracture	Fatigue, overload	Thread stress concentration	Use optimized screw design
Prosthesis fracture	Stress concentration	Load transfer via screw head	Occlusal control, material selection
Misfit-related stress	Inaccurate fabrication	Screw compensates misfit	Passive fit verification
Wear/chipping	Material properties	Indirect effect via load distribution	Material and occlusion control

## Data Availability

No new data were created or analyzed in this study. Data sharing is not applicable to this article.

## Supplementary Materials

The following supporting information can be downloaded at: https://www.mdpi.com/article/10.3390/ma19112212/s1, Table S1: Representative mechanical properties of restorative materials relevant to titanium base–free MUA restorations; Table S2: Representative quantitative parameters related to screw-joint mechanics.

## Abbreviations

The following abbreviations are used in this manuscript:

MUA	Multi-unit abutment
CAD	Computer-aided design
CAM	Computer-aided manufacturing
CAD/CAM	Computer-aided design and computer-aided manufacturing
PEEK	Polyetheretherketone
PMMA	Polymethyl methacrylate
Co-Cr	Cobalt–chromium
DLC	Diamond-like carbon
Ti	Titanium
Ti-base	Titanium base
Ncm	Newton centimeter
DESS	Dental Smart Solutions
