# Supplementary material for: Titanium Base-Free Multi-Unit Abutment Connections: A Critical Review of Prosthetic Screw Design, Biomechanical Behavior, and Clinical Performance
Source: Materials (Basel). 2026 May 24;19(11):2212. doi: 10.3390/ma19112212 (PMC13258801; doi:10.3390/ma19112212)
Supplement: Supplementary file 1 [file materials-19-02212-s001.zip › materials-4313187-supplementary.pdf]

Supplementary Table S1. Representative mechanical properties of restorative materials relevant to titanium base–free MUA restorations.

Material	Elastic modulus	Flexural strength / strength	Fracture toughness	Hardness	Biomechanical relevance
Zirconia	~180–210 GPa	~600–1200 MPa	~2–10 MPa·m^1/2	~1100–1300 HV / ~11–13 GPa	High rigidity; greater stress transfer to screw and implant–abutment interface
Cobalt-chromium alloy	~170–230 GPa	~600–1000 MPa	Not commonly reported for dental frameworks	~300–450 HV	Rigid framework material; efficient load distribution but limited damping
Titanium alloy	~105–120 GPa	~800–950 MPa	Not commonly reported for dental frameworks	~300–380 HV	Lower stiffness than Co-Cr; favorable ductility
PEEK	~3–4 GPa; higher when reinforced	~140–180 MPa; higher when reinforced	~3–5 MPa·m^1/2	~20–35 HV	Stress-dampening behavior but lower hardness and wear resistance
PMMA	~2–3.5 GPa	~60–110 MPa	~1–2 MPa·m^1/2	~15–25 HV	Low stiffness; suitable mainly for provisional or transitional prostheses
CAD-CAM resin composite	~8–20 GPa	~120–250 MPa	~1–3 MPa·m^1/2	~50–100 HV	Intermediate stiffness; more repairable but lower rigidity than zirconia/metals

Values are representative ranges and may vary according to manufacturer, composition, processing method, aging protocol, and testing method. HV, Vickers hardness; PEEK, polyetheretherketone; PMMA, polymethyl methacrylate.

Supplementary Table S2. Representative quantitative parameters related to screw-joint mechanics.

Parameter	Representative value/range	Biomechanical relevance
Torque converted into preload	~10–15% of applied torque	Only a small fraction of torque becomes effective clamping force
Torque lost to friction	~85–90% of applied torque	Friction strongly influences preload generation
Early preload loss due to settling	~2–10% shortly after tightening	Supports retightening protocols
Recommended torque for direct-to-MUA systems	~10–25 Ncm, system-dependent	Manufacturer-specific torque protocols must be followed
Common fatigue testing load	~100–200 N	Frequently used in laboratory fatigue studies
Common fatigue testing cycles	~10^6 cycles	Often used to approximate one year of chewing simulation
Reverse torque / preload loss	Highly variable	Depends on screw geometry, coating, fit, torque protocol, and loading
Micromovement	System- and method-dependent	Depends on connection geometry, fit, and loading direction

Values are representative background parameters and should not be interpreted as pooled estimates for titanium base–free MUA restorations. MUA, multi-unit abutment.
